# Completeness of reporting acupuncture interventions for chronic obstructive pulmonary disease: Review of adherence to the STRICTA statement

**DOI:** 10.12688/f1000research.22843.3

**Published:** 2020-11-20

**Authors:** Carles Fernández-Jané, Mireia Solà-Madurell, Mingkun Yu, Changhao Liang, Yutong Fei, Mercè Sitjà-Rabert, Gerard Úrrutia

**Affiliations:** 1 School of Health Science Blanquerna, Ramon Llull University, Barcelona, Spain; 2 Global Research on Wellbeing (GRoW) Research Group, Ramon Llull University, Barcelona, Spain; 3 Centre for Evidence-Based Chinese Medicine, Beijing University of Chinese Medicine, Beijing, China; 4 Institut d'Investigació Biomèdica Sant Pau (IIB Sant Pau). CIBER de Epidemiología y Salud Pública (CIBERESP), Barcelona, Spain

**Keywords:** Quality of Reporting, Acupuncture, COPD

## Abstract

**Background:** The completeness of reporting  of acupuncture interventions is critical to ensure the applicability and reproducibility of acupuncture clinical trials. In the past, different publications have evaluated the completeness of reporting of acupuncture interventions for different clinical situations, such as knee osteoarthritis, neurological diseases or cancer. However, this has not been done for acupuncture trials for chronic obstructive pulmonary disease (COPD).

**Objective: **To assess the completeness of reporting of acupuncture interventions in trials for COPD.

**Methods:** A total of 11 English and Chinese databases were screened up until May 2019 for randomised or quasi-randomised control trials of acupuncture for COPD. The STRICTA checklist was used to determine the quality of the reporting of acupuncture interventions.

**Results:** A total of 28 trials were included in our review. Out of the 16 STRICTA checklist subitems analysed, only 4 were considered appropriately reported in more than 70% of the trials, while 7 were correctly reported in less than 30%.

**Conclusion: **The adherence to STRICTA guidelines of acupuncture trials for COPD is suboptimal, and future efforts need to be addressed to improve the completeness of reporting.

## Introduction

Recent systematic reviews have assessed acupuncture’s efficacy for chronic obstructive pulmonary disease (COPD) ^1– 3^. These reviews have concluded that, even though acupuncture could have some benefits, the risk of bias of the included trials is too high to draw any strong conclusion. Risk of bias is certainly a critical aspect in randomised control trials; however, to be able to adequately assess sources of bias, complete information needs to be reported.

Complete and clear information regarding clinical trial methodology is not only essential to adequately assess health research but also for its applicability and reproducibility. This is especially important in complex interventions, such as acupuncture, that can be practiced in many different styles and variations. Aspects such as point selection, depth of the insertion, stimulation method and response sought, which may be very different between practitioners, could have an impact on the therapeutic effect ^4^. Therefore, to be able to replicate an acupuncture intervention in clinical practice or reproduce it in another trial, it is necessary to fully describe how it is applied.

The STandards for Reporting Interventions in Controlled Trials of Acupuncture (STRICTA) guidelines were created to improve the completeness of reporting in acupuncture trials and facilitate transparency in published reports ^5^. These guidelines were updated in 2010 as an extension of the CONsolidated Standards Of Reporting Trials (CONSORT) guidelines ^6^ and consist of 6 key items and 17 subitems addressing aspects such as acupuncture rationale, needling details, treatment regime, other components of the treatment, practitioner background and details about the control or comparator.

Although there is evidence that STRICTA guidelines have helped to improve acupuncture’s reporting, there is still a lot of room for improvement ^7^. This has also been seen in recent publications for some specific conditions such as neurological diseases ^8– 10^ or cancer ^11^, concluding that reporting is still suboptimal in these conditions. However, to our knowledge, there is currently no publications regarding the completeness of reporting of acupuncture interventions in COPD trials.

Evaluating the adherence of acupuncture clinical trials for COPD to SRICTA guidelines is crucial to detect underreported subitems and therefore highlight current deficiencies. This will help to improve the reporting of future trials and facilitate their applicability in clinical practice and the reproducibility of future research.

The aim of this study is to comprehensibly evaluate the degree of completeness of reporting for each STRICTA item in randomised trials using acupuncture.

## Methods

### Study selection

In this study, we used the results of our previous systematic review, which included randomised or quasi-randomised trials using filiform needle acupuncture for COPD ^2^. Published studies were comprehensively searched in the following databases from their inception to May 2019: Cochrane Central Register of Controlled Trials (CENTRAL), Medline, Embase, CINAHL, AMED (Ovid), PEDro, PsycINFO, CNKI, VIP, Wanfang, and Sino-Med. Detailed descriptions of the inclusion criteria, information sources, search strategies and study selections are published elsewhere ^2^ and the protocol is available at http://www.crd.york.ac.uk/PROSPERO/display_record.asp?ID=CRD42014015074.

### Data collection process

Data from each trail was extracted independently by two reviewers (CFJ, MSM, MY and CHL) using a standardised data extraction form, and disagreements were solved by consensus.

For data collection and STRICTA assessment, a specific extraction table with instructions of how to assess each of the 16 STRICTA subitems was created by the authors ( *Extended data* ^12^). This extraction table was tested with pilot data of 3 papers to solve disagreements on its understanding and ensure its usability. The data of the pilot test was included in the final analysis.

Our aim was to assess the acupuncture interventions, and therefore, the 17th STRICTA subitem “precise description of the control or comparator” was not considered. However, we did include the 16th STRICTA subitem “rationale of the chosen comparator”, since it is critical to justify which component of the acupuncture treatment is being assessed.

Since some STRICTA subitems refer to multiple aspects (e.g., “names of points used” subitem refers not only to the name or location of points but also to if they are used unilaterally or bilaterally), besides considering items just as reported or not reported, we also considered partially reported items and recorded the reasons for there being. This was done to provide more detailed information regarding the aspects that should be improved in the reporting of future trials. Partial reporting was also considered when the authors reported information in other sections, such as the introduction or the discussion. Although some subitems were considered that could potentially be “not applicable” (NA) for some pragmatic designs, none of the trials required this.

### Data analysis

A descriptive analysis was used to summarise the results using percentages and absolute numbers.

## Results

### Number of publications and characteristics

**Figure 1.**
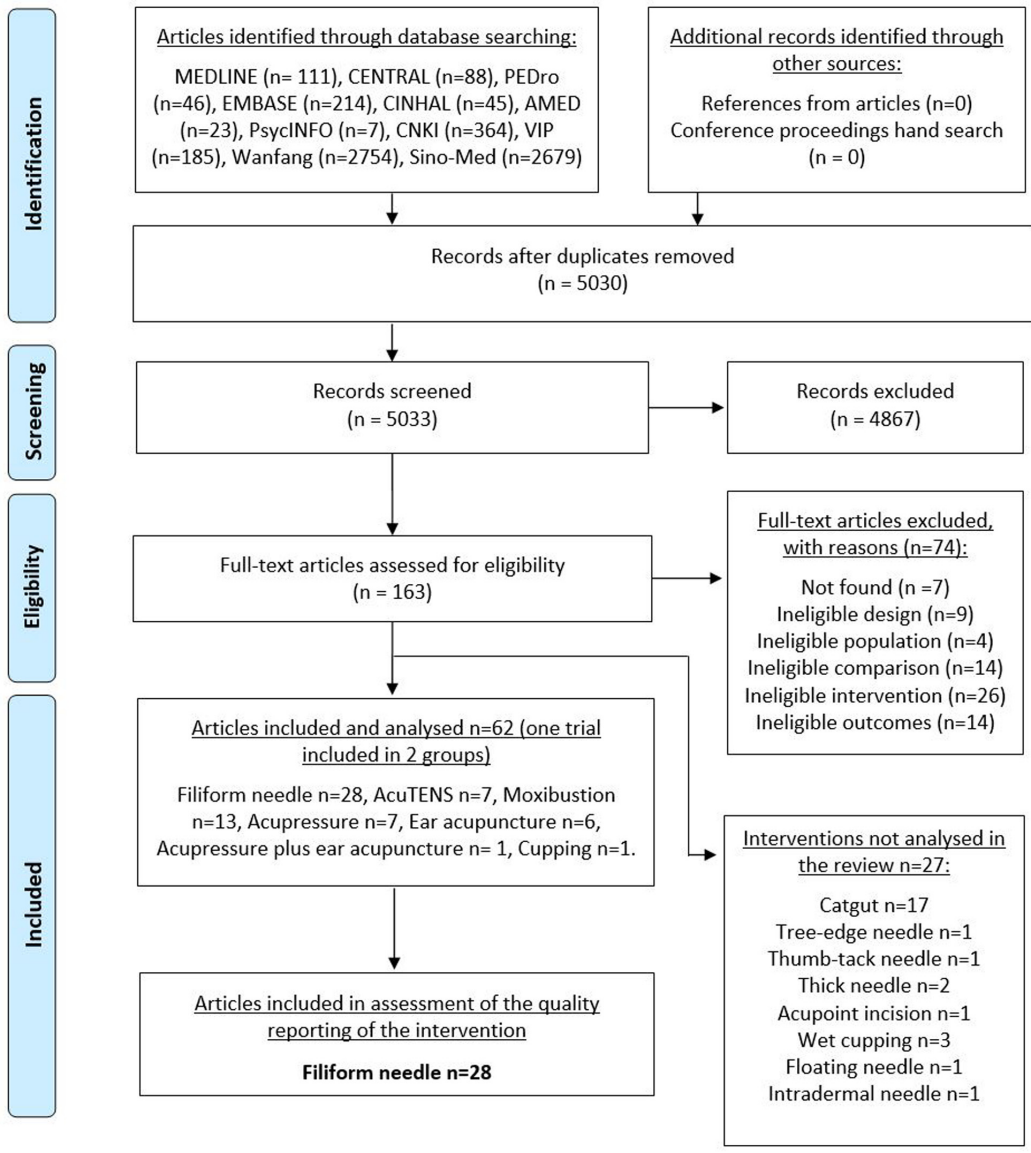
Flow diagram.

In our systematic review, we screened all 5030 unduplicated titles and abstracts retrieved, and obtained 166 full text articles. Finally, we included 28 trials using filiform needles for COPD ( Figure 1). Of those, only 6 were published in English-language journals, 1 in a German-language journal and 21 in Chinese-language journals. Details regarding the study characteristics and inclusion process have been published elsewhere ^2^.

### Completeness of reporting

Out of the 16 STRICTA checklist subitems analysed, only 4 were considered appropriately reported in more than 70% of the trials; style of acupuncture, variation extent, retention time and frequency and duration of treatment sessions. We also found that 7 other subitems were correctly reported in less than 30% of the trials; depth of insertion, needle type, number of treatment sessions, details of other interventions administered, setting and context of treatment, description of participating acupuncturists and rationale for the control.

**Figure 2.**
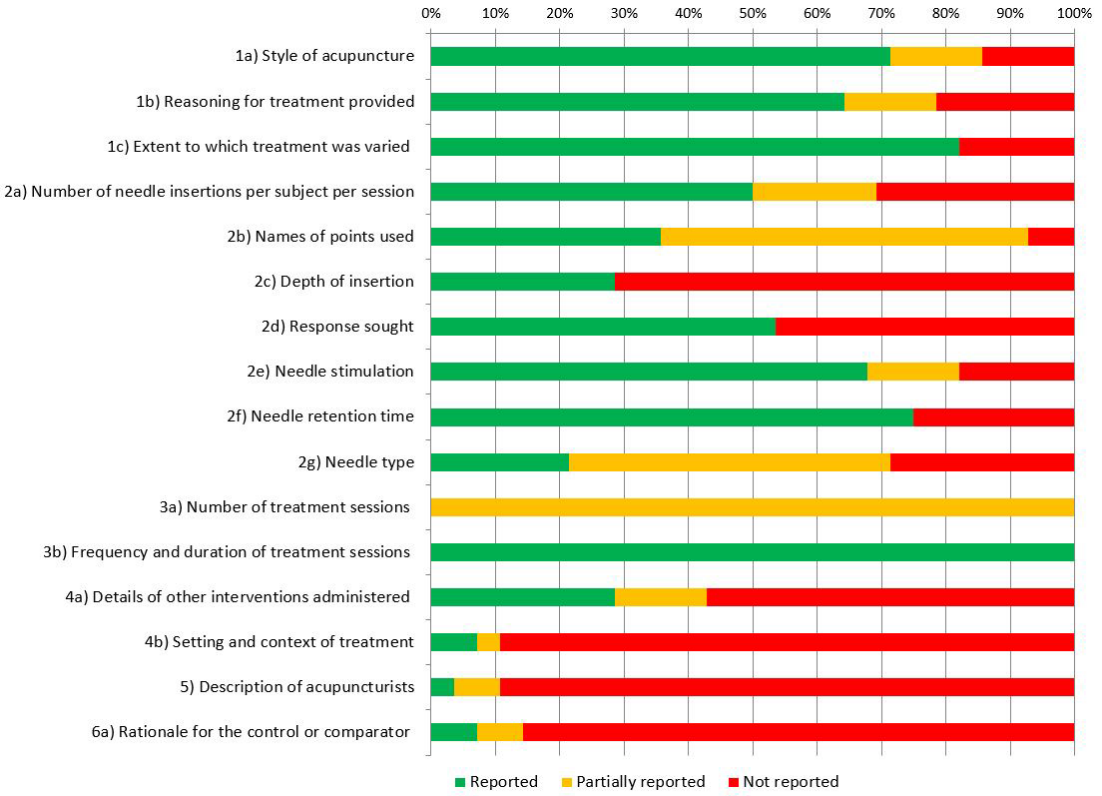
Summary of completeness of reporting according to STRICTA guidelines.

Ratings for STRICTA domains are summarized in Figure 2. Details for each trial are shown in Table 1, including reasons for considering partial reporting.

**Table 1.** Completeness of reporting according to STRICTA guidelines.

STRICTA Items		Reported, % (n)	Not reported, % (n)	Partially reported, % (n) Reason, % (n)
1) Acupuncture rationale	1a) Style of acupuncture	71.4 (20)	14.2 (4)	14.2 (4) Not reported in the correct section, 14.2 (4)
	1b) Reasoning for treatment provided, based on historical context, literature sources, and/or consensus methods, with references where appropriate	64.2 (18)	21.4 (6)	14.2 (4) Not reported in the correct section, 14.2 (4)
	1c) Extent to which treatment was varied	82.1 (23)	17.8 (5)	0 (0)
2) Details of needling	2a) Number of needle insertions per subject per session	46.4 (13)	28.5 (8)	17.8 (5) Do not mention the number of needles, 17.8 (5)
	2b) Names (or location if no standard name) of points used (uni/bilateral)	35.7 (10)	7.14 (2)	57.14 (16) Do not mention uni or bilateral insertion, 53 (15) Not all point locations are described, 3.5 (1)
	2c) Depth of insertion, based on a specified unit of measurement or on a particular tissue level	28.5 (8)	71.4 (20)	0 (0)
	2d) Responses sought	53.5 (15)	46.4 (13)	0 (0)
	2e) Needle stimulation	67.8 (19)	17.8 (5)	14.2 (4) Manual stimulation is mentioned but the specify the method is not, 10.7 (3) Electrical stimulation is mentioned but not parameters used, 3.5 (1)
	2f) Needle retention time	75 (21)	25 (7)	0 (0)
	2g) Needle type	21.4 (6)	28.5 (8)	50 (14) Material is not reported, 25 (7) Manufacturer is not reported, 32.1 (9) Diameter and length are not reported, 10.7 (3)
3) Treatment regime	3a) Number of treatment sessions	0 (0)	0 (0)	100 (28) The actual number of treatments received is not reported or not clear, 100 (28)
	3b) Frequency and duration of treatment sessions	100 (28)	0 (0)	0 (0)
4) Other components of treatment	4a) Details of other interventions administered to the acupuncture group	28.5 (8)	57.1 (16)	14.2 (4)
	4b) Setting and context of treatment, including instructions to practitioners, and information and explanations to patients	7.1 (2)	89.2 (25)	3.5 (1)
5) Practitioner background	5) Description of participating acupuncturists	3.5 (1)	89.2 (25)	7.1 (2) Years of experience not mentioned, 7.1 (2)
6) Control or comparator interventions	6a) Rationale for the control or comparator	7.1 (2)	85.7 (24)	7.1 (2) Not reported in the correct section: 7.1 (2)

STRICTA, STandards for Reporting Interventions in Clinical Trials of Acupuncture.

***Acupuncture rationale.*** Acupuncture rationale was considered adequately reported in 20 trials (71%) regarding acupuncture style, 18 trials (64%) regarding reasoning of the treatment and 24 trials (85%) regarding treatment variation. Trials classified with partial reporting mentioned acupuncture style (3 trials) and reasoning (4 trials) in the introduction section but not in the methods section.

***Details of needling.*** Adequate reporting of needling details was very heterogenous along all 4 subdomains. Best reported subdomains were “needle retention time” (21 trials, 75%) and “needle stimulation” (19 trials, 67%). Worse reported items were “name of the points” (10 trials, 35%), “depth of insertion” (8 trials, 28%) and “needle type” (6 trials, 21%). Partial reporting was observed in great proportion in “name of acupuncture points” (16 trials, 57%) being the main reason not describing if points were used unilaterally or bilaterally. Regarding the 46% of the trials classified with a partial reporting in the “needle type” item, there was missing information about the needle manufacturer (32.1%, 9 trials), material (25%, 7 trials) and length and diameter (10.7%, 3 trials).

***Treatment regime.*** While “frequency and duration of the treatment sessions” was considered adequately reported for all trials (100%), the “number of treatment sessions” was considered completely reported in none of them (0%). Although this might seem a contradiction, since the number of treatment sessions can be calculated from the treatment regime, in STRICTA, the number of treatment sessions does not only include the planned number of sessions but also the actual number of treatments received. This information was missing or considered not clear in all trials.

***Other components of treatment.*** Other components of treatment were one of the poorer described items. “Details of other interventions administrated” was only reported in 8 trials (28%) and “settings and context of treatments”, which refers to “instructions to practitioners that might modify their normal practice, for example, prescribing or proscribing explanations to patients about their diagnosis”, were only reported in 2 trials (7.1%).

***Practitioner background.*** This item was only addressed completely in 1 trial (3.5%), and only 2 more trials (7.1%) partially reported it without stating practitioner’s years of experience.

***Control or comparator.*** The “rational of the chosen comparator” was only correctly described in 2 trials (7.1%), while in 2 more trials (7.1%), this was mentioned in the introduction but not in the methods section.

## Discussion

We found important limitations in the completeness of reporting of acupuncture interventions in trials for COPD, especially regarding “depth of insertion”, “needle type”, “number of treatment sessions”, “details of other interventions administered”, “setting and context of treatments”, “description of acupuncturist” and “rationale for the control or comparator” with less than 30% of the trials reporting them completely.

Recently, several similar studies have been published. Lu *et al.* ^11^ and Hughes *et al*. ^13^ used STRICTA to evaluate trials with cancer patients, and Wei *et al.* used STRICTA to evaluate trials with stroke patients ^8^. Although they all concluded that reporting should be improved, there were also some differences. While Hughes *et al.* found better reporting regarding details of needling and treatment regimen, other reviews found lower reporting on these subitems, especially regarding number of needles per session and depth of insertion. Poor reporting on “details of other interventions administered”, “description of acupuncturist” and “rationale for the control or comparator” subitems was found in all studies.

The differences mentioned above could be due to several reasons. First, Lu’s and Weis’s reviews, as well as our own, included Chinese-language trials, while Hugs’ study included only English-language trials. Trials published in English-language journals seem to have greater completeness of reporting than those in Chinese-language journals, according to Lu’s review. However, Wei *et al.* found better reporting of the subitems “treatment reasoning” and “response sought” in Chinese journals and better reporting on “practitioner’s background” in English journals.

Second, since STRICTA does not clearly specify how items should be judged, authors might have used different criteria. For example, regarding the subitem “number of treatment sessions”, the STRICTA statement says that “the actual number of treatments received by participants should be reported in the Results section” not only the planned ones. Whereas in our review, we did not consider that this subitem was fully reported unless this information was explicitly stated; others might have been more permissive. Also, the criteria to consider proper reporting on “other components of treatment” might vary widely between reviewers.

Third, sometimes information might have been reported in sections such as the introduction and the discussion. While some authors might not have given much importance to this, we decided to take it into consideration.

To try to improve the adherence to reporting guidelines several strategies have been proposed including training on the use of the guidelines, improving understanding, encouraging adherence, checking adherence and providing feedback, and the involvement of experts. Unfortunately, the effectiveness of many of those interventions is still unknown ^14^.

### Strengths and limitations

To our knowledge, this is the first study to assess the completeness of reporting of acupuncture interventions for COPD. We included all acupuncture trials for COPD published until May 2019 with no language restriction, which is important since we only found 6 acupuncture trials published in English. Also, we did not only assess if STRICTA subitems were adequately reported or not but also analysed partial reporting and its reasons, which might be more helpful for authors to realise what specific information is currently missing.

Limitations of this study include that the STRICTA guidelines are not a rating scale; therefore, there are no clear indications of how to judge each subitem and when to consider it fully reported. This issuer must be addressed in the future by developing a proper completeness of reporting assessment tool for acupuncture interventions. To minimise this problem, each item was assessed by two reviewers independently, and a standardised extraction form was developed to unify reviewers’ criteria. Also, it would have been interesting to compare the completeness of reporting depending on the language of publication, so we could explore differences in journal standards. However, due to the low number of non-Chinese-language publications found (1 in German and 6 in English), we decided not to do so. Finally, STRICTA guidelines are specific for the filiform needle acupuncture technique and are not suitable to assess other interventions. Therefore, we did not include trials using only moxibustion, acupressure or transcutaneous electrical nerve stimulation.

## Conclusion

The completeness of reporting of acupuncture interventions in COPD trials according to STRICTA guidelines is suboptimal. Strategies for improving the understanding of the guides for authors, reviewers and journal editors are needed, as well as to improve its implementation.

## Data availability

### Underlying data

Figshare: Raw data file, https://doi.org/10.6084/m9.figshare.11999970.v1 ^15^.

### Extended data

Figshare: Extended data 1 Extraction form, https://doi.org/10.6084/m9.figshare.11999994.v1 ^12^.

Data is available under a Creative Commons Attribution 4.0 International license (CC-BY 4.0).
