# Supplementary material for: Completeness of reporting acupuncture interventions for chronic obstructive pulmonary disease: Review of adherence to the STRICTA statement
Source: F1000Res. 2020 Nov 20;9:226. Originally published 2020 Apr 2. [Version 3] doi: 10.12688/f1000research.22843.3 (PMC7713895; doi:10.12688/f1000research.22843.3)
Supplement: Peer review history

# Peer review: Reviewer response for version 3

**DOI:** 10.5256/f1000research.30829.r75127

**Published:** 2020-12-01

**Authors:** Erik Cobo

**Affiliations:** 1 Statistics and Operations Research Department, Polytechnic University of Catalonia, Barcelona, Spain

Thanks for considering most of my suggestions. I think the paper is now much clearer and probably more reprodicible.

Is the work clearly and accurately presented and does it cite the current literature?

Yes

If applicable, is the statistical analysis and its interpretation appropriate?

Yes

Are all the source data underlying the results available to ensure full reproducibility?

No

Is the study design appropriate and is the work technically sound?

Yes

Are the conclusions drawn adequately supported by the results?

Yes

Are sufficient details of methods and analysis provided to allow replication by others?

Yes

Reviewer Expertise:

Clinical trials; Reporting Guidelines; meta-research;  research waste

I confirm that I have read this submission and believe that I have an appropriate level of expertise to confirm that it is of an acceptable scientific standard.

# Peer review: Reviewer response for version 3

**DOI:** 10.5256/f1000research.30829.r75126

**Published:** 2020-11-23

**Authors:** David Blanco

**Affiliations:** 1 Physiotherapy Department, Universitat Internacional de Catalunya, Barcelona, Spain

I am happy with this new version of the manuscript. It is clear and well-reported. Congratulations on your work and thank you for the opportunity to review it!

Is the work clearly and accurately presented and does it cite the current literature?

Partly

If applicable, is the statistical analysis and its interpretation appropriate?

Yes

Are all the source data underlying the results available to ensure full reproducibility?

Yes

Is the study design appropriate and is the work technically sound?

Yes

Are the conclusions drawn adequately supported by the results?

Yes

Are sufficient details of methods and analysis provided to allow replication by others?

Yes

Reviewer Expertise:

Journal policies, peer review, reporting guidelines

I confirm that I have read this submission and believe that I have an appropriate level of expertise to confirm that it is of an acceptable scientific standard.

# Peer review: Reviewer response for version 2

**DOI:** 10.5256/f1000research.30190.r73827

**Published:** 2020-11-13

**Authors:** David Blanco

**Affiliations:** 1 Physiotherapy Department, Universitat Internacional de Catalunya, Barcelona, Spain

Thank you for allowing me to review this manuscript. The subject is the evaluation of the degree of adherence to STRICTA guideline items in the context of acupuncture trials. Overall, I find the manuscript attractive and well-written.

 I hope the authors find the following suggestions helpful:

**General comments**

- Despite being a term commonly used in the literature, I would suggest authors to avoid using “quality of reporting”, as “quality” is an ambiguous and problematic word, and to use “completeness of reporting”, which is clearer and more connected to reporting guidelines ^1^ ^2^.

**Abstract**

- If you include the sentence: “Out of the 16 STRICTA checklist subitems analysed, only 3 were considered appropriately reported in more than 70% of the trials, while 7 were correctly reported in less than 40%.” you would also need to include this information in the Results section (perhaps as a first paragraph of the “Quality of reporting” subsection). As a reader, I would appreciate to have in the main body of the article some sort of “overall summary” of the completeness of the included papers and not just the results for each separate item.

**Introduction**

- In the last sentence of the first paragraph, you seem to imply that the risk of bias of a certain study and its quality of reporting have no connection. In fact, complete and transparent reporting is what makes it possible for a reviewer to assess the risk of bias of a study. I believe this point deserves to be reflected in the paper.
- Importantly, the fact that you state, “The aim of this study is to comprehensibly evaluate the adherence to STRICTA guidelines of trials using acupuncture for COPD”, could make the reader think that you are going to use the percentage of completeness per trial as the unit of interest. However, you are analysing the percentage of completeness per STRICTA item. I would therefore suggest to reformulate the study aim to avoid confusion: “The aim of this study is to comprehensibly evaluate the degree of completeness of reporting for each STRICTA item in randomised trials using acupuncture” or something similar.

**Methods**

- It would be helpful that you explain in a few words what was the structure of the standardized data extraction form and what you were interested in looking at.
- Were the three papers you used for the pilot also included in the final analysis? This should be mentioned.

**Discussion**

- In the first paragraph of the discussion, it would be good to mention in brackets the criteria that you chose to highlight these underreported items (% of adequate reporting less than X%?)
- The sentence “The differences between studies could be due to several reasons” should be placed at the beginning of the next paragraph as it is heavily connected with it. For example, “The differences between the studies we mentioned above could be due to several reasons.”
- Concerning the (very appropriate) point you make in the fourth paragraph, you could also mention that RGs are originally just guidance for writing and they are not intended to be evaluation forms despite everyone uses them as such ^3^. 
- I would also better argue why you took into consideration (and considered as “partially reported” instead of fully reported) those items that were reported in a different section. I guess that when you say section you refer to the main 4 sections (IMRaD) – perhaps you could make it clearer in the methods.
- For me, it is not enough to say “Strategies for improving the understanding of the guides for authors, reviewers, and journal editors are needed, as well as to improve its implementation”. You should definitely include a paragraph in the Discussion section reflecting on what kind of strategies targeting authors, peer reviewers, or journal editors could work in the particular context of acupuncture journals (or more generally, if you prefer so). In the introduction, you mentioned, “This will help to improve the reporting of future trials and facilitate their applicability in clinical practice and the reproducibility of future research.” However, I believe that it is not enough to point out that there are reporting issues without reflecting on what kind of strategies should journals follow to turn things around. As you probably know, some recent experiments are testing out different editorial strategies ^4^ ^5^ ^6^ ^7^ ^8^ as well as different scoping and systematic reviews/surveys exploring this issue.

Is the work clearly and accurately presented and does it cite the current literature?

Partly

If applicable, is the statistical analysis and its interpretation appropriate?

Yes

Are all the source data underlying the results available to ensure full reproducibility?

Yes

Is the study design appropriate and is the work technically sound?

Yes

Are the conclusions drawn adequately supported by the results?

Yes

Are sufficient details of methods and analysis provided to allow replication by others?

Yes

Reviewer Expertise:

Journal policies, peer review, reporting guidelines

I confirm that I have read this submission and believe that I have an appropriate level of expertise to confirm that it is of an acceptable scientific standard, however I have significant reservations, as outlined above.

## References

[1] : Relation of completeness of reporting of health research to journals' endorsement of reporting guidelines: systematic review. *BMJ*.2014;348: 10.1136/bmj.g3804 g3804 10.1136/bmj.g3804 24965222PMC4070413

[2] : Scoping review on interventions to improve adherence to reporting guidelines in health research. *BMJ Open*.2019;9(5) : 10.1136/bmjopen-2018-026589 10.1136/bmjopen-2018-026589 PMC652799631076472

[3] : Reporting guideline checklists are not quality evaluation forms: they are guidance for writing. *Health Sci Rep*.2020;3(2) : 10.1002/hsr2.165 e165 10.1002/hsr2.165 32373717PMC7196677

[4] : A randomised controlled trial of an Intervention to Improve Compliance with the ARRIVE guidelines (IICARus). *Res Integr Peer Rev*.2019;4: 10.1186/s41073-019-0069-3 12 10.1186/s41073-019-0069-3 31205756PMC6560728

[5] : Impact of an online writing aid tool for writing a randomized trial report: the COBWEB (Consort-based WEB tool) randomized controlled trial. *BMC Med*.2015;13: 10.1186/s12916-015-0460-y 221 10.1186/s12916-015-0460-y 26370288PMC4570037

[6] : Effect of an editorial intervention to improve the completeness of reporting of randomised trials: a randomised controlled trial. *BMJ Open*.2020;10(5) : 10.1136/bmjopen-2020-036799 10.1136/bmjopen-2020-036799 PMC723954132430454

[7] : Effect of using reporting guidelines during peer review on quality of final manuscripts submitted to a biomedical journal: masked randomised trial. *BMJ*.2011;343: 10.1136/bmj.d6783 d6783 10.1136/bmj.d6783 22108262PMC3222149

[8] : Impact of a web-based tool (WebCONSORT) to improve the reporting of randomised trials: results of a randomised controlled trial. *BMC Med*.2016;14(1) : 10.1186/s12916-016-0736-x 199 10.1186/s12916-016-0736-x 27894295PMC5126856

# Response

**Published:** 2020-11-15

**Authors:** Carles Fernández

**Affiliations:** School of Health Sciences Blanquerna, Spain

We thank you for your time and comments on the manuscript. Please find below a point by point response to them:

**General comments**

- Despite being a term commonly used in the literature, I would suggest authors to avoid using “quality of reporting”, as “quality” is an ambiguous and problematic word, and to use “completeness of reporting”, which is clearer and more connected to reporting guidelines ^1^ ^2^.

Response: We agree with the suggestion and have changed “quality of reporting” for “completeness of reporting”.

**Abstract**

- If you include the sentence: “Out of the 16 STRICTA checklist subitems analysed, only 3 were considered appropriately reported in more than 70% of the trials, while 7 were correctly reported in less than 40%.” you would also need to include this information in the Results section (perhaps as a first paragraph of the “Quality of reporting” subsection). As a reader, I would appreciate to have in the main body of the article some sort of “overall summary” of the completeness of the included papers and not just the results for each separate item.

Response: We have now added the information at the beginning of the results section.

**Introduction**

- In the last sentence of the first paragraph, you seem to imply that the risk of bias of a certain study and its quality of reporting have no connection. In fact, complete and transparent reporting is what makes it possible for a reviewer to assess the risk of bias of a study. I believe this point deserves to be reflected in the paper.

Response: We agree with the reviewers comments and therefore, we modified our last sentence to show that risk of bias and its quality of reporting are connected

 “Risk of bias is certainly a critical aspect in randomised control trials; however, to be able to adequately assess sources of bias, complete information needs to be reported.”

- Importantly, the fact that you state, “The aim of this study is to comprehensibly evaluate the adherence to STRICTA guidelines of trials using acupuncture for COPD”, could make the reader think that you are going to use the percentage of completeness per trial as the unit of interest. However, you are analysing the percentage of completeness per STRICTA item. I would therefore suggest to reformulate the study aim to avoid confusion: “The aim of this study is to comprehensibly evaluate the degree of completeness of reporting for each STRICTA item in randomised trials using acupuncture” or something similar.

Response: We have reformulated the aim of the study to avoid confisions as the reviewer suggested.

**Methods**

- It would be helpful that you explain in a few words what was the structure of the standardized data extraction form and what you were interested in looking at.

Response: The structure of the standardized data extraction table is fully available as extended data

- Were the three papers you used for the pilot also included in the final analysis? This should be mentioned.

Response: Yes, he pilot data extraction was included in the analysis. This has been clarified in the data collection section

**Discussion**

- In the first paragraph of the discussion, it would be good to mention in brackets the criteria that you chose to highlight these underreported items (% of adequate reporting less than X%?)

Response: This has been now described

 “We found important limitations in the quality of reporting of acupuncture interventions in trials for COPD, especially regarding “depth of insertion”, “needle type”, “number of treatment sessions”, “details of other interventions administered”, “setting and context of treatments”, “description of acupuncturist” and “rationale for the control or comparator” with less than 30% of the trials reporting them completely.”

- The sentence “The differences between studies could be due to several reasons” should be placed at the beginning of the next paragraph as it is heavily connected with it. For example, “The differences between the studies we mentioned above could be due to several reasons.”

Response: This has been corrected

- Concerning the (very appropriate) point you make in the fourth paragraph, you could also mention that RGs are originally just guidance for writing and they are not intended to be evaluation forms despite everyone uses them as such ^3^. 

Response: This is explained in the limitations section “Limitations of this study include that the STRICTA guidelines are not a rating scale; therefore, there are no clear indications of how to judge each subitem and when to consider it fully reported. This issuer must be addressed in the future by developing a proper quality of reporting assessment tool for acupuncture interventions.”

- I would also better argue why you took into consideration (and considered as “partially reported” instead of fully reported) those items that were reported in a different section. I guess that when you say section you refer to the main 4 sections (IMRaD) – perhaps you could make it clearer in the methods.

Response: It was already explained in the methods section “Partial reporting was also considered when the authors reported information in other sections, such as the introduction or the discussion.”. However we have now clarified also in the discussion section “Third, sometimes information might have been reported in sections such as the introduction and the discussion.”

- For me, it is not enough to say “Strategies for improving the understanding of the guides for authors, reviewers, and journal editors are needed, as well as to improve its implementation”. You should definitely include a paragraph in the Discussion section reflecting on what kind of strategies targeting authors, peer reviewers, or journal editors could work in the particular context of acupuncture journals (or more generally, if you prefer so). In the introduction, you mentioned, “This will help to improve the reporting of future trials and facilitate their applicability in clinical practice and the reproducibility of future research.” However, I believe that it is not enough to point out that there are reporting issues without reflecting on what kind of strategies should journals follow to turn things around. As you probably know, some recent experiments are testing out different editorial strategies ^4^ ^5^ ^6^ ^7^ ^8^ as well as different scoping and systematic reviews/surveys exploring this issue.

Response: A new paragraph in the discussion section about the topic has been added

 “To try to improve the adherence to reporting guidelines several strategies have been proposed including training on the use of the guidelines, improving understanding, encouraging adherence, checking adherence and providing feedback, and the involvement of experts. Unfortunately, the effectiveness of many of those interventions is still unknown ^14^”

# Peer review: Reviewer response for version 1

**DOI:** 10.5256/f1000research.25221.r71710

**Published:** 2020-10-06

**Authors:** Erik Cobo

**Affiliations:** 1 Statistics and Operations Research Department, Polytechnic University of Catalonia, Barcelona, Spain

I read this paper and I think it could be accepted provided authors correct my three major suggestions and address my minor suggestions.

**Major suggestions:**

- Please revise the numbers in your flow diagram and add explanations (maybe in a legend) about your more important labels, such us ineligible population/comparison/… Regarding the numbers, please note that the box “records screened” has more records than the previous one. Or than 62 >< 28+7+13+7+6+1+1=63. Please also note that studies are not the same unit as articles. Please, unify.
- Please consider to re-write your conclusions clarifying which recommendations could potentially improve quality in the future. I mean, recommendations to STRICTA authors; recommendations to journals; recommendations to reviewers, recommendations to authors; and so on.
- Please, allow access to your data to other scientists. Please, consider a public repository.

**Minor suggestions:**

- As you stated, “the STRICTA guidelines are not a rating scale”. However, you are repeating through the text that you are attempting to “assess the quality of reporting”. I wonder if you should also highlight the need to further develop reporting guidelines to provide such a tool to assess the quality of reporting. Should you speak about the completeness of reporting? I definitively like much more your statement “evaluate the adherence to Stricta guidelines”.
- In the introduction, I wonder if you should clarify that “risk of bias” applies to the study; and “quality of reporting” to the paper.
- Also in the introduction, you use the terms "replication" and "reproducibility". I would like you to clarify your meanings, perhaps with some references. For example, are you following the definition from Goodman, Fannelly & Ioanidis in “What does research reproducibility mean?” ^1^
- Please, further specify how reviewers “worked in pairs”? Did they split the sample? Did both pairs of reviewers analyze some papers?
- Please, consider to explain the pilot process and how did you “ensure” its usability.

Is the work clearly and accurately presented and does it cite the current literature?

Yes

If applicable, is the statistical analysis and its interpretation appropriate?

Yes

Are all the source data underlying the results available to ensure full reproducibility?

No

Is the study design appropriate and is the work technically sound?

Yes

Are the conclusions drawn adequately supported by the results?

Yes

Are sufficient details of methods and analysis provided to allow replication by others?

Yes

Reviewer Expertise:

RCTs, reporting guidelines, causal modeling, meta-researcch

I confirm that I have read this submission and believe that I have an appropriate level of expertise to confirm that it is of an acceptable scientific standard, however I have significant reservations, as outlined above.

## References

[1] : What does research reproducibility mean?. *Science Translational Medicine*.2016;8(341) : 10.1126/scitranslmed.aaf5027 10.1126/scitranslmed.aaf5027 27252173

# Response

**Published:** 2020-10-11

**Authors:** Carles Fernández

**Affiliations:** School of Health Sciences Blanquerna, Spain

We thank you for your time and comments on the manuscript. Please find below a point by point response to them:

**Major suggestions:**

**Please revise the numbers in your flow diagram and add explanations (maybe in a legend) about your more important labels, such us ineligible population/comparison/… Regarding the numbers, please note that the box “records screened” has more records than the previous one. Or than 62 >< 28+7+13+7+6+1+1=63. Please also note that studies are not the same unit as articles. Please, unify.**

As it is already explained in the figure, one trial included two interventions, that is why the total number of studies included is 62 but the summation of all the interventions is 63.

Studies has been changed for articles in the diagram

**Please consider to re-write your conclusions clarifying which recommendations could potentially improve quality in the future. I mean, recommendations to STRICTA authors; recommendations to journals; recommendations to reviewers, recommendations to authors; and so on.**

Conclusions have been re-written to include the mentioned recommendations

“The quality of reporting of acupuncture interventions in COPD trials according to STRICTA guidelines is suboptimal. Strategies for improving the understanding of the guides for authors, reviewers and journal editors are needed, as well as to improve its implementation.”

**Please, allow access to your data to other scientists. Please, consider a public repository.**

The data is already public and available in the “data availability” section.

**Minor suggestions:**

**As you stated, “the STRICTA guidelines are not a rating scale”. However, you are repeating through the text that you are attempting to “assess the quality of reporting”. I wonder if you should also highlight the need to further develop reporting guidelines to provide such a tool to assess the quality of reporting. Should you speak about the completeness of reporting? I definitively like much more your statement “evaluate the adherence to Stricta guidelines”.**

We agree on highlighting the need of developing a reliable tool for assessing the quality of reporting and this has been added in the discussion section.

We would prefer not to change the nomenclature as the real the objective of the study is to assess the quality of reporting, and using the adherence to current reporting guidelines is the method to do so. We believe that introducing the term “completeness of the reporting” may confuse reader.

**In the introduction, I wonder if you should clarify that “risk of bias” applies to the study; and “quality of reporting” to the paper.**

This has been clarified in the introduction section

“Risk of bias of bias of the studies is certainly a critical aspect in randomised control trials; however, the quality of reporting of the published papers is also a key point.”

**Also in the introduction, you use the terms "replication" and "reproducibility". I would like you to clarify your meanings, perhaps with some references. For example, are you following the definition from Goodman, Fannelly & Ioanidis in “What does research reproducibility mean?” ^1^**

Here replication and reproducibility are used in a common language sense and the meaning is clarified at the end of the introduction section:

“Therefore, to be able to replicate an acupuncture intervention in clinical practice or reproduce it in another trial, it is necessary to fully describe how it is applied.”

We believe that using the terms “methods reproducibility”, “Results reproducibility” or “inferential reproducibility” might not clarify but confuse the reader.

**Please, further specify how reviewers “worked in pairs”? Did they split the sample? Did both pairs of reviewers analyze some papers?**

Every paper was analysed by two reviewers, this has been now clarified in the methods section:

“Data from each trail was extracted independently by two reviewers (CFJ, MSM, MY and CHL) using a standardised data extraction form, and disagreements were solved by consensus.”

**Please, consider to explain the pilot process and how did you “ensure” its usability.**

This has been now clarified in the “data collection process” section.

“This extraction table was tested with pilot data of 3 papers to solve disagreements on its understanding and ensure its usability.”
